# Mef2c Regulates Transcription of the Extracellular Matrix Protein Cartilage Link Protein 1 in the Developing Murine Heart

**DOI:** 10.1371/journal.pone.0057073

**Published:** 2013-02-26

**Authors:** Marie M. Lockhart, Elaine E. Wirrig, Aimee L. Phelps, Angela V. Ghatnekar, Jeremy L. Barth, Russell A. Norris, Andy Wessels

**Affiliations:** 1 Department of Regenerative Medicine and Cell Biology, Medical University of South Carolina, Charleston, South Carolina, United States of America; 2 The Heart Institute, Cincinnati Children’s Hospital Medical Center, Cincinnati, Ohio, United States of America; 3 Division of Rheumatology and Immunology, Medical University of South Carolina, Charleston, South Carolina, United States of America; Northwestern University, United States of America

## Abstract

Cartilage Link Protein 1 (Crtl1) is an extracellular matrix (ECM) protein that stabilizes the interaction between hyaluronan and versican and is expressed in endocardial and endocardially-derived cells in the developing heart, including cells in the atrioventricular (AV) and outflow tract (OFT) cushions. Previous investigations into the transcriptional regulation of the Crtl1 gene have shown that Sox9 regulates Crtl1 expression in both cartilage and the AV valves. The cardiac transcription factor Mef2c is involved in the regulation of gene expression in cardiac and skeletal muscle cell lineages. In this study we have investigated the potential role of Mef2c in the regulation of ECM production in the endocardial and mesenchymal cell lineages of the developing heart. We demonstrate that the Crtl1 5′ flanking region contains two highly conserved Mef2 binding sites and that Mef2c is able to bind to these sites *in vivo* during cardiovascular development. Additionally, we show that Crtl1 transcription is dependent on Mef2c expression in fetal mitral valve interstitial cells (VICs). Combined, these findings highlight a new role for Mef2c in cardiac development and the regulation of cardiac extracellular matrix protein expression.

## Introduction

Cartilage Link Protein 1 (Crtl1; also known as Hyaluronan and Proteoglycan Binding Protein 1- Hapln1) is a glycoprotein found in the extracellular matrix (ECM) of multiple tissues, including cartilage, brain and heart [1]–[3]. Crtl1 is involved in the formation and stabilization of proteoglycan and hyaluronan aggregates [4], [5] and is important for preventing aggregate degradation by proteases such as members of the ADAMTS and MMP families [5]–[7]. Loss of Crtl1 results in impairment of growth and development of several tissues, including the cartilage, heart, and central nervous system [1]–[3]. Crtl1 null mice are characterized by craniofacial abnormalities and shortened long bones, abnormalities attributed to a reduction in aggrecan within the cartilage resulting in an inability of chondrocytes to differentiate and hypertrophy [3]. Cardiac malformations seen in Crtl1 knockout mice include muscular ventricular septal defects, atrioventricular septal defects, and thin myocardium [2]. Crtl1 knockout mice die perinatally which has been attributed to compromised lung development [3].

The mechanisms governing regulation of ECM protein expression are complex and involve many signaling pathways [8]. Recent studies have shown that similarities exist between the transcriptional programs that regulate bone formation and the formation of cardiac valves during development (reviewed in [9]). In the developing bone and the AV valves, Crtl1 has previously been shown to be directly regulated by the transcription factor, Sox9 [10], [11]. Conditionally deleting Sox9 in the valves of the developing mouse heart does, however, not completely abolish Crtl1 expression, indicating that the expression of Crtl1 in the developing valves is not solely dependent on Sox9 [12].

During endochondral bone development, Sox9 has been demonstrated to regulate chondrocyte hypertrophy by transactivating the Col10a1 gene along with the transcription factor Mef2c [13]. The expression of both Sox9 and Mef2c within the cartilage is required for chondrocyte hypertrophy [14], [15]. Cartilage-specific loss of Sox9, independently or in conjunction with Mef2c, results in delayed chondrocyte hypertrophy and shortened long bones, producing a dwarfism phenotype similar to that seen in the Crtl1 knockout mouse [13], [15]. Within the heart, the Mef2 family of transcription factors has long been associated with regulation of myocardially-expressed genes, including cardiac alpha-actin and alpha-myosin heavy chain [16]–[18]. Mef2c knockout mice die around ED9.5 due to defects in heart looping and chamber formation. Interestingly, Mef2c null embryos fail to form endocardial cushions and have little cardiac jelly, indicating that the establishment of ECM may be affected [19].

In this study we have tested the hypothesis that Mef2c acts as a transcriptional regulator of Crtl1 expression in the developing heart. Immunohistochemical and *in situ* hybridization expression analyses demonstrate that during cardiac development Crtl1 and Mef2c are co-expressed in endocardial and mesenchymal cells. We identified two highly conserved Mef2 binding sites within the 5′ upstream sequences of the Crtl1 promoter. Using chromatin immunoprecipitation (ChIP) we show that Mef2c is able to bind to the conserved Mef2 binding sites *in vivo* during cardiovascular development. Finally, we show that, using an *in vitro* approach, in fetal mitral valve interstitial cells (VICs), Crtl1 transcription is dependent on Mef2c expression. The data presented here reveal a new role for Mef2c in cardiac development, specifically related to the transcriptional regulation of extracellular matrix protein expression.

## Experimental Procedures

### Sequence Alignment

The 5′ upstream sequences of the Crtl1 promoter for mouse, rat, and human genes were aligned using the web-based tool Kalign [20]. All sequences are available at NCBI, the mouse sequence AF139572, rat sequences NM019189 and CH473955, and human sequences NM001884 and NT006713 were used for the alignment.

### Ethics Statement

This study was carried out in strict accordance with the recommendations in the Guide for the Care and Use of Laboratory Animals of the National Institutes of Health. The protocol (AR#2464) was approved by the Institutional Animal Care & Use Committee (IACUC) at the Medical University of South Carolina. Wildtype C57BL6/J embryos were collected from timed pregnant dams and staged according to Theiler (1989) before being processed for immunohistochemistry, *in situ* hybridization, or Chromatin Immunoprecipitation as described below.

### Immunohistochemistry

Embryos were fixed in 4% paraformaldehyde (PFA) for 4 hours. Tissue processing, hematoxylin/eosin staining, and immunohistochemistry were performed as previously described [21]. Antibodies used included: mouse monoclonal anti-Crtl1 (Developmental Studies Hybridoma Bank, 9/30/8-A-4) [22], [23], rabbit polyclonal anti-Mef2c (Sigma, HPA005533), and rabbit polyclonal anti-Sox9 (Santa Cruz, sc-20091). For fluorescent detection of the primary antibodies, Donkey anti-mouse FITC (Jackson Immunoresearch, #715-095-150), and Donkey anti-rabbit TRITC (Jackson Immunoresearch, #711-025-152) were used. Immunofluorescently stained sections were imaged using the Zeiss AxioImager 2.0 microscope system.

### In Situ Hybridization

Section in situ hybridization was performed as described [24]. Probes were made by isolating total RNA from mouse embryonic tissues, using the RNeasy minikit (Qiagen #74104), and synthesizing cDNA using the Stratascript first strand kit (Stratagene #200420). Following cDNA synthesis, a PCR product was amplified using primers for Cartilage Link Protein 1 (Ctrl1) sense (5′-tggaccaggacgcagtgatt-3′) and antisense (5′-gcagcggtcatagcccagaa-3′) with the addition of Sp6 or T7 polymerase promoters. The Crtl1 mRNA probe was synthesized and labeled from PCR product using the DIG RNA Sp6/T7 labeling kit (Roche #11-175-025-910). The tissues were de-waxed in xylenes and rehydrated through an ethanol series, and then digested with 15 ug/mL of proteinase K. Following proteinase K digestion, the tissues were treated with 0.2% glycine and post-fixed in 4%PFA/0.2% gluteraldehyde solution. The tissues were then hybridized with the RNA DIG-labeled probe overnight at 68°C. After thorough washing on the second day, tissues were blocked in 10% sheep serum/1% blocking reagent (Roche #11-093-274-910). The hybridized probe was visualized using Immuno BCIP/NBT substrate (MP Biochemicals #980771).

### DNA Precipitation

#### Oligo design

Sense and anti-sense fifty-nucleotide oligos from the Crtl1 promoter sequence containing the potential Mef2 consensus binding sites and flanking sequence were obtained from Operon with a biotin tag on the 5′ end of the sense strand (see Table 1). The sense and anti-sense oligos were annealed to form double stranded DNA oligos. To disrupt Mef2c binding, mutated oligos were designed where the Mef2 consensus site from −707 to −698 was mutated from 5′-ctataaataa-3′ to 5′-ctatagcgaa-3′ and the Mef2 consensus site from −922 to −913 was mutated from 5′-ttataaataa-3′ to 5′-ttatagcgaa-3′. Positive and negative control-oligos were designed with three Mef2 consensus sites from the muscle creatine kinase gene (known to bind with Mef2) or with mutated sites with random intervening sequence. DNA affinity precipitation assays were performed as described by others [25], [26]. Ripa buffer extracts of wildtype embryonic hearts ED12.5–14.0 (approximately 200 µg of protein), 20 µg poly DI/DC, 100 µL of 10x binding buffer (40 mM KCl, 15 mM HEPES pH 7.9, 1 mM EDTA, 0.5 mM DTT), and 5% glycerol in a final sample volume of 1 mL were precleared with streptavidin agarose beads (Invitrogen #15942-050). Following preclearing to remove background, the samples were incubated with 30 pM of annealed oligos overnight at 4°C. Streptavidin agarose beads were then reintroduced to bind the biotin tag of the annealed oligos. Subsequently, the beads were thoroughly washed in 1x TBE buffer, then 1X binding buffer, and lastly PBS. Protein/DNA oligo complexes were eluted from the beads by boiling in 4X sample buffer at 95°C for 5 minutes. Eluted protein was run on a 4–20% Tris-glycine gel (Invitrogen, #EC6025), then subsequently transferred to a nitrocellulose membrane (Invitrogen, #LC2001), blocked in 5% dry milk/1%TBST, and probed with primary antibody against Mef2c (Santa Cruz, sc-13266). The secondary antibody used was Donkey anti-goat HRP (Santa Cruz, sc-2033). ECL Advanced reagents were used to detect antibody binding (Amersham/GE Healthcare, #2135). Three independent experiments were performed.

**Table 1:** DNA Precipitation Oligonucleotides.

Oligonucleotide	Sequence
Positive control Mef2 sense	5′ [biotin]-tcgctctaaaaataaccctcaagctctaaaaataaccctgtcactctaaaaataaccctgag
Positive control Mef2 antisense	5′-ctcagggttatttttagagtgacagggttatttttagagcttgagggttatttttagagcga
Negative control Mef2 sense	5′ [biotin]- tcgctaaagcgaaccctcaagctaaagcgaaccctgtcactctaaagcgaaccctgag
Negative control Mef2 antisense	5′-ctcagggttcgctttagagtgacagggttcgctttagagcttgagggttcgctttagagcga
Mef2 wildtype site −707 to −698 sense	5′ [biotin]- tcccccttgcattcctcactctataaataaactcaggttcttaggcacta
Mef2 wildtype site −707 to −698 antisense	5′-tagtgcctaagaacctgagtttatttatagagtgaggaatgcaaggggga
Mef2 mutated site −707 to −698 sense	5′ [biotin]- tcccccttgcattcctcactctatagcgaaactcaggttcttaggcacta
Mef2 mutated site −707 to −698 antisense	5′-tagtgcctaagaacctgagtttcgctatagagtgaggaatgca-aggggga
Mef2 wildtype site −922 to −913 sense	5′ [biotin]-tatactctccctcgagttataaataaatgtctatttgttcaggaggaggtt
Mef2 wildtype site −922 to −913 antisense	5′-aacgcctcctgaacaaatagacatttatttataactcgagggagagtata
Mef2 mutated site −922 to −913 sense	5′ [biotin] tatactctccctcgagttatagcgaaatgtctatttgttcaggaggcgtt
Mef2 mutated site −922 to −913 antisense	5′-aacgcctcctgaacaaatagacatttcgctataactcgagggagagtata

### Chromatin Immunoprecipitation

Wildtype embryonic hearts at stages ED10.5–11.5 were collected in cold PBS and then incubated in 1% Formaldehyde in PBS for 10 minutes at room temperature. Formaldehyde cross-linking was stopped by adding 10X Glycine to a final concentration of 1X and incubating at room temperature for 5 minutes. Tissue was spun at 4°C at 5,000rcf for 5 minutes and the remaining tissue pellet was rinsed twice in ice-cold PBS. The tissue was then resuspended in an SDS Lysis Buffer containing a Protease Inhibitor Cocktail (Upstate EZ-Chip, #17–371), sheared by passing through a 28-gauge needle, and then sonicated. Chromatin Immunoprecipitation was performed four times using the Upstate EZ-Chip protocol for suspension cells with 5 µg of the goat polyclonal antibody, anti-Mef2c (Santa Cruz, sc-13266) and 5 µg of normal goat IgG (Santa Cruz, sc-2028) as an isotype control. Using the same protocol, Chromatin Immunoprecipitation was also performed two times with 1 µg of the rabbit polyclonal antibodies: anti-Mef2c (Sigma, HPA00553) and anti-Sox9 (Santa Cruz, sc-20095) and 1 µg of normal rabbit IgG (Caltag, #10500C) as an isotype control. Immunoprecipitated DNA was PCR amplified using primers designed to amplify the mouse Crtl1 promoter sequence spanning both the Mef2 binding sites at −707 to −698 (5′-ctataaataa-3′) and at −913 to −923 (5′-ttataaataa-3′); forward primer (5′-ccaatcaaaggtggctctgt-3′) and reverse primer (5′-acccagattgcttgttttgc-3′). The same immunoprecipitated DNA was also PCR amplified to asses binding to the Sox9 consensus site of the mouse Crtl1 promoter using the forward primer (5′-atcctcggatcaggacctct-3′) and the reverse primer (5′-aaacccaccaaacagaaacg-3′).

### Plasmids

The mouse Crtl1 promoter (Crtl1) from −979 to +26 was PCR amplified from genomic mouse DNA using the forward primer (5′-ccaaaccccttggctactcaaggc-3′) and the reverse primer (5′-cacttagctgggagctggag-3′). The promoter was then cloned into a pGL3-Basic luciferase reporter vector (Promega, #E1751) between the KpnI and HindIII restriction enzyme sites. Mutations were made to the Crtl1 construct at each Mef2 binding site using PCR Site-directed Mutagenesis and then cloned into pGL3-Basic.The Mef2 consensus site from −707 to −698 was mutated from 5′-ctataaataa-3′ to 5′-ctatagcgaa-3′ (Crtl1-Mutant1) and the Mef2 consensus site from -922 to -913 was mutated from 5′-ttataaataa-3′ to 5′-ttatagcgaa-3′ (Crtl1-Mutant2).The mouse Mef2c expression construct and Mef2-Engrailed dominant negative construct were provided by Dr. Eric Olson, University of Texas Southwestern Medical Center [15].

### Mitral VIC Isolation

Mitral valves from HH40 chick embryos were removed and digested with 400 µL of trypsin for 30 minutes. Mitral VICs were cultured in 100 mm cell culture dishes at 37°C with 5% CO_2_ in M199 medium (Gibco, #11150-059) with 1% chick serum, 1% penicillin/streptomycin, and 0.1% Insulin/Transferrin/Selenium (ITS).

### NIH3T3 Cell Culture

NIH3T3 cells were cultured at 37°C with 5% CO_2_ in high glucose DMEM (Fisher, #SH3028501) with 10% fetal bovine serum (Fisher, #SH3007001), 1% penicillin/streptomycin, and 4 mM L-Glutamine (Fisher, #SH3003401).

### Luciferase Assay

Primary chick mitral VICs were plated after 2–4 passages at a cell density of 1×10^5^ and transfected in triplicate with the following constructs: mCrtl1, mMef2c, Mef2-Engrailed, and pGL3-Basic as a control. NIH3T3 cells were plated at a cell density of 2×10^5^ and transfected in triplicate with Crtl1, Crtl1-Mutant1, Crtl1-Mutant2, mMef2c, and pGL3-Basic as a control. In both experiments with primary chick Mitral VICs and NIH3T3 cells, pIRES-GFP and pSV-β-galactosidase (Promega #E1081) vectors were co-transfected to determine transfection efficiency (pIRES-GFP) and to normalize the relative light units from the luciferase assay measurements. Transfections were performed using the Fugene reagent (Roche, #11-815-091-001) with a Fugene:DNA ratio of 6:2. After 48 hours, transfected mitral VIC extracts were prepared using reporter lysis buffer (Promega #E3971). To measure the luciferase activity, 20 µL of cell extract was added to 100 µL of Luciferase Assay Reagent (Promega #E1500) and the light produced was measured on a Monolight 2010 luminometer. Measurement of β-galactosidase activity in cell extracts was performed using the Promega β-galactosidase Enzyme Assay (Promega #E2000), and the A420 nm readings were used to standardize the luciferase assay values. Three replicates were used in each experiment and two independent experiments were performed. In the result section, the data obtained are represented as the average fold change for all replicates (n = 6) relative to controls. The error bars represent the standard error of the mean. An unpaired, two-tailed, Student’s T-test was used to test for significance.

## Results

### The Cartilage Link Protein Promoter Contains Two Highly Conserved Mef2 Binding Sites

Previous investigations by others into the regulation of the rat Crtl1 gene have shown that the upstream promoter region contains an A-T rich element that is conserved between mouse, rat, and human and has a high degree of similarity to A-T rich elements found in the muscle creatine kinase promoter [27]. This A-T rich element is able to activate Crtl1 transcription in response to serum and can bind to an unidentified 32 kDa protein in electromobility shift assays using chondrocyte cell nuclear extracts [27]. It has been hypothesized that this protein could be a homeodomain containing protein or a MADS domain transcription factor [27].

**Figure 1:**
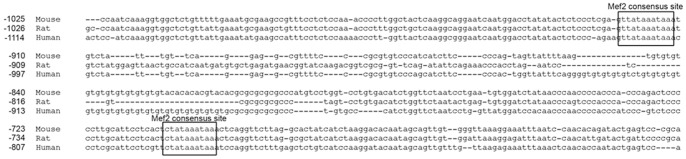
Sequence alignment of the mouse, rat, and human Crtl1 (Hapln1) promoters. The mouse, rat, and human Crtl1 genes, plus 1000 bp of the upstream promoter, were aligned using the web-based tool Kalign. Using this alignment, two conserved Mef2 consensus sites were identified at positions −698 to −707 and −913 to −923.

The Mef2 transcription factors are members of the MADS domain family of transcription factors that bind to A-T rich sequences and regulate expression of multiple cardiac and skeletal muscle specific genes [28]. To determine whether Mef2 transcription factor binding sites are present in the Crtl1 promoter, approximately 1 kb of the human, rat, and mouse Crtl1 5′-flanking region were aligned using the software Kalign [20]. A search of the aligned sequences for the Mef2 consensus sequence CTA(A/T)_4_TAG [29] identified two potential Mef2 binding sites that were evolutionarily conserved between the mouse, rat, and human Crtl1 promoters (Figure 1). In the mouse Crtl1 promoter, one Mef2 binding site is positioned at −707 to −698 (5′-ctataaataa-3′), designated as Mef2 Site 1, while the other is positioned at −913 to −923 (5′-ttataaataa-3′), designated as Mef2 Site 2. Interestingly, the Mef2 site at position −913 to −923 lies within the previously described A-T rich element of the Crtl1 promoter [27].

### Crtl1 and Mef2c are Expressed by Endocardial and Mesenchymal Cells of the Developing Heart

**Figure 2:**
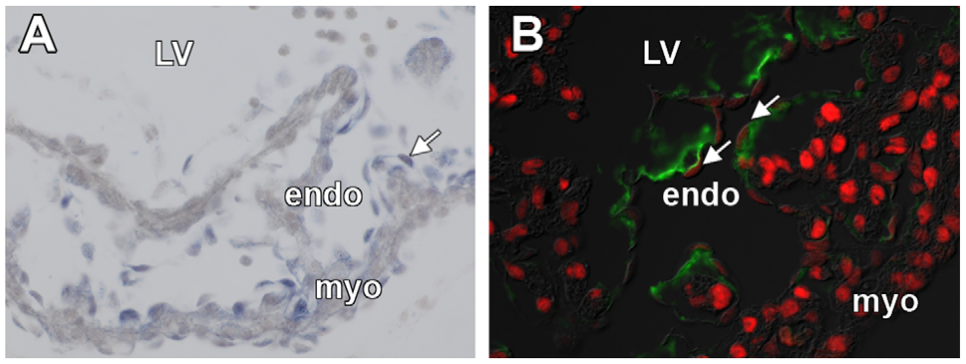
Crtl1, Mef2c, and Sox9 expression in the ventricular endocardium at ED10.5. Crtl1 mRNA, Crtl1 protein, and Mef2c protein expression in the ventricular endocardium at ED10.5. (A) Crtl1 mRNA (blue staining, white arrow) is synthesized by ventricular endocardial cells at ED10.5. (B) Crtl1 protein expression (green) is observed in the ventricular cardiac jelly between the endocardial and myocardial cell layers. Mef2c protein expression (red) is observed in the nuclei of both the ventricular myocardium and ventricular endocardium (white arrows indicate endocardial Mef2c expression). endo = endocardium, myo = myocardium, LV = left ventricle.

Crtl1 is expressed in the extracellular matrix of the developing outflow tract cushions, AV cushions, and in the cardiac jelly that resides between the ventricular endocardium and developing trabeculae [2]. *In situ* hybridization demonstrates that at ED10.5 Crtl1 mRNA is synthesized by ventricular endocardial cells (Figure 2A). The Crtl1 protein is located in the ECM between the endocardium and ventricular myocardium (Figure 2B). To determine whether Mef2c is also expressed by ventricular endocardial cells, immunohistochemistry was performed on sections of ED10.5–11.0 hearts, which localized Mef2c protein in the nuclei of ventricular endocardial cells that also synthesize Crtl1 mRNA (Figure 2B).

**Figure 3:**
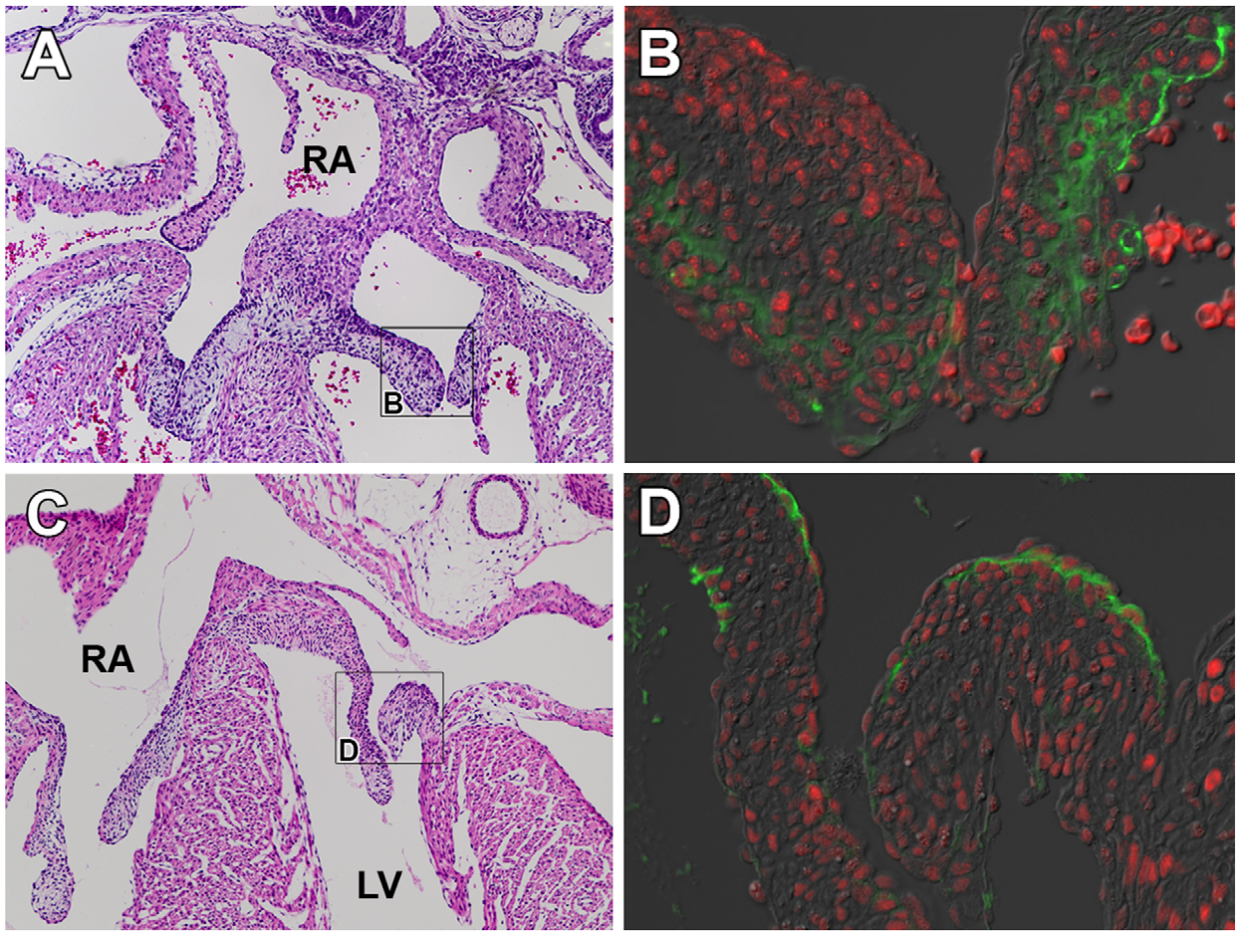
Crtl1 and Mef2c expression in the AV junction at ED14.5 and ED17.5. (A) Atrioventricular junction in an H&E stained ED14.5 specimen. (B) Immunofluorescent staining of Crtl1 (green) shows Crtl1 is expressed in the ventricular aspect of the leaflets of the mitral valve at ED14.5, Mef2c (red, panel B) is also expressed throughout the leaflets of the mitral valve. (C) Atrioventricular junction in an H&E stained ED17.5 specimen. (D) Crtl1 is expressed sub-endocardially on the atrial aspects of the mitral valve leaflets at ED17.5 and Mef2c (red, panel F) is expressed in both the mesenchyme and endocardial lining of the leaflets, colocalizing with Crtl1 protein expression (green, panels D).

Crtl1 is also expressed in the developing AV cushions and in the leaflets of the AV valves as they mature. In the developing AV valves at ED14.5, Crtl1 is expressed in the mesenchyme of the AV valve leaflets (Figure 3B) and, as the valve matures, Crtl1 becomes restricted to the endocardial lining of the leaflets by ED17.5 (Figure 3D). Mef2c is also expressed in the maturing AV valve leaflets. At ED14.5 and 17.5, Mef2c is expressed throughout the leaflet mesenchyme as well as in the endocardial lining of the leaflets (Figure 3B,D).

### Mef2 Binds to the Cartilage Link Protein Promoter

**Figure 4:**
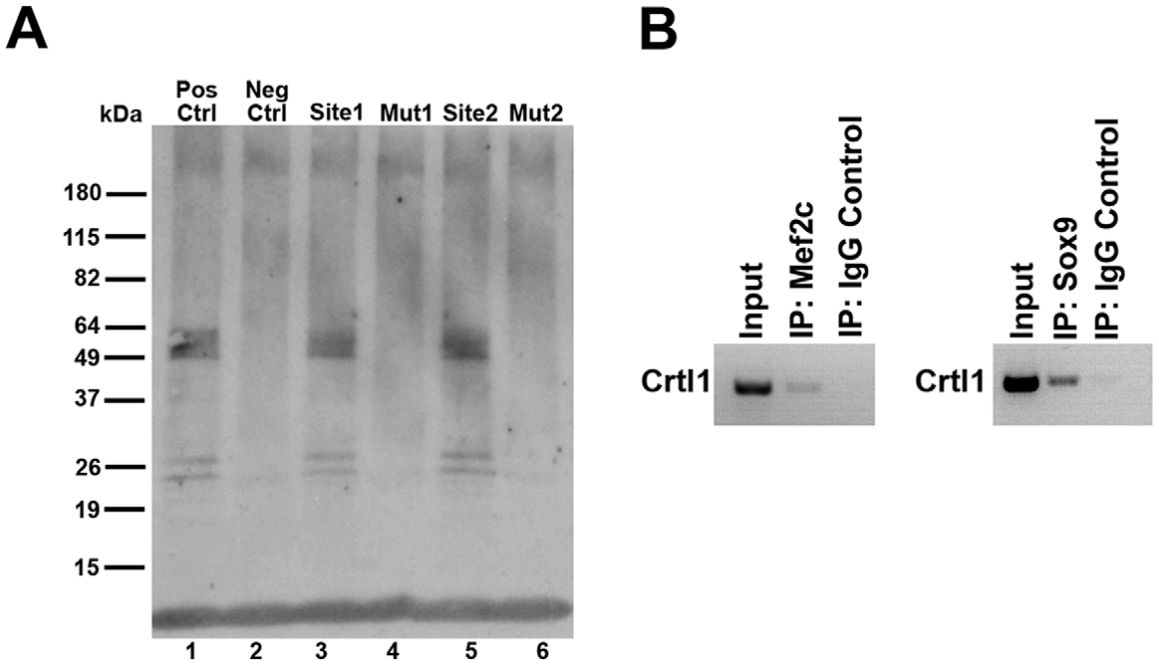
Mef2c binds to the Crtl1 Promoter in the developing heart. (A) Immunoblot for Mef2c protein. Lane 1, Mef2c binding to control Mef2 consensus sites from the muscle creatine kinase gene. Lane 2, negative control oligo with mutated Mef2 sites fails to bind to Mef2c. Lanes 3 and 5, Mef2c interacts with the Mef2 consensus sites in the Crtl1 promoter region at −913 to −923 (Site 1, Lane 3) and at −698 to −707 (Site 2, Lane 5). Lanes 4 and 6, mutation of the Mef2 consensus sites in the Crtl1 promoter region blocks Mef2 binding at both consensus sites. (B) Mef2c and Sox9 bind the Crtl1 Promoter *in* *vivo*. Chromatin Immunoprecipitation (ChIP) was performed using embryonic hearts stage ED10.5–11.0. PCR of the DNA input, Mef2c immunoprecipitate, and IgG control precipitate was performed using primers flanking both Mef2 consensus sites of the Crtl1 promoter. For the Sox9 ChIP, the initial DNA input, Sox9 immunoprecipitate, and IgG control precipitate were PCR amplified using primers specific for the Sox9 consensus site on the Crtl1 promoter.

To determine whether the Mef2 consensus sites identified in the 5′ flanking region of the Crtl1 promoter do in fact bind to Mef2c, a DNA affinity precipitation assay [25], [26] was performed using biotin-tagged oligos corresponding to each of the putative Mef2 sites. The Mef2 consensus site at −913 to −923 is referred to as Mef2 site 1 and the consensus site −698 to −707 is referred to as Mef2 site 2 (Figure 4A). Biotin-tagged oligos with mutations at each Mef2 site (Mut1 and Mut2) were also made by inserting a –gcg- sequence within the consensus site. Western blot analysis of the biotin-tagged DNA/protein complexes demonstrates that Mef2c protein binds to both Mef2 consensus sites identified in the promoter and that mutation of these sites results in loss of Mef2c bound protein.

In order to confirm the findings of the DNA precipitation assay, and to detect binding of Mef2c to the Crtl1 promoter *in vivo*, chromatin immunoprecipitation (ChIP) was performed on embryonic heart tissue using a Mef2c antibody to retrieve Mef2c bound DNA. As an internal positive control, antibodies to Sox9 were also used for ChIP, as it has been demonstrated by others that Sox9 regulates Crtl1 in the AV cushions and also binds to the Crtl1 promoter [12], [30]. PCR amplification of the immunoprecipitate using primers spanning both Mef2 consensus sites in the Crtl1 promoter showed that Mef2c binds to the promoter in embryonic heart tissue (Figure 4B).

### Mef2c Activates Crtl1 Transcription in Fetal Mitral VICs and NIH3T3 Cells

**Figure 5:**
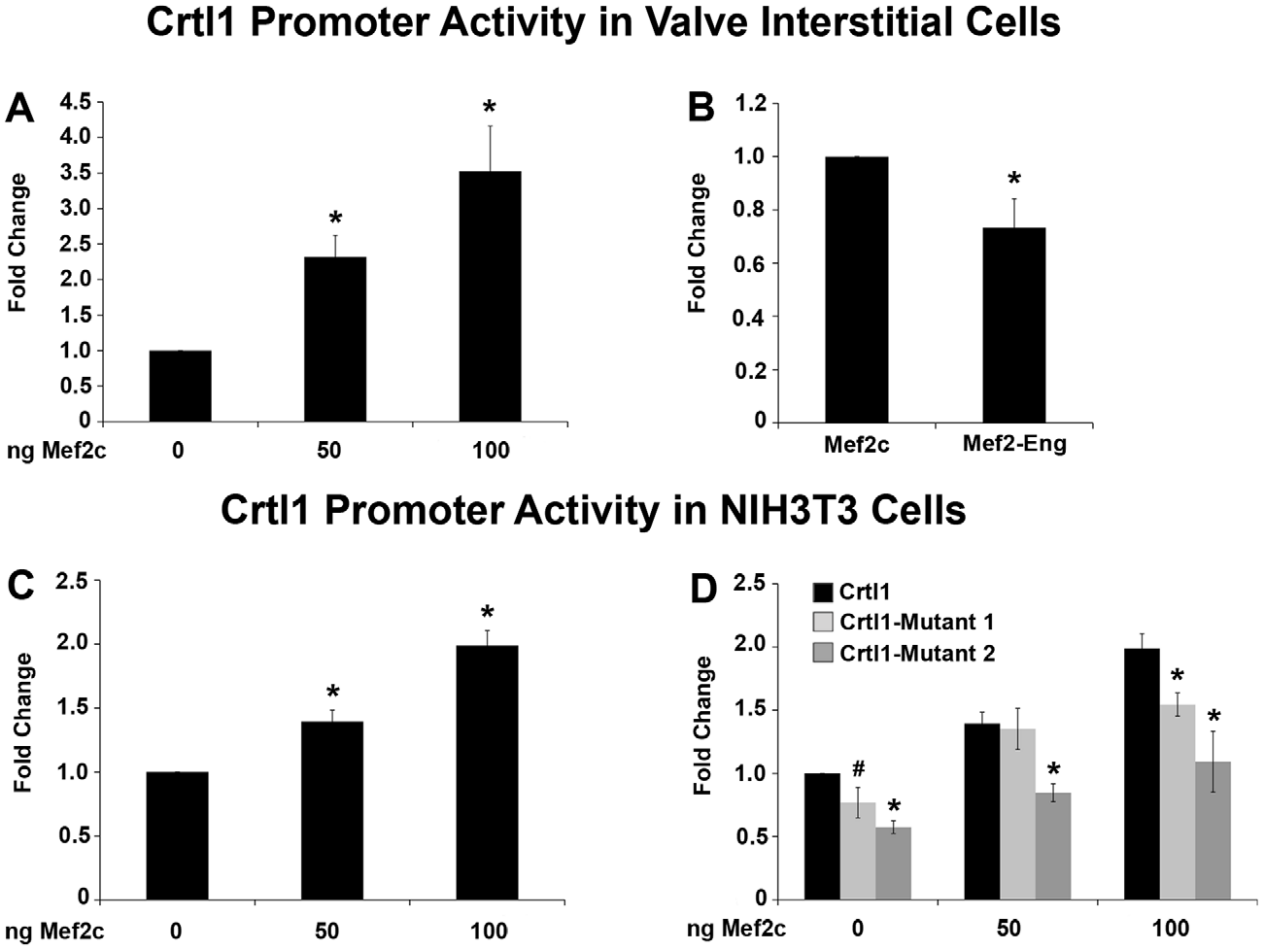
Crtl1 promoter activity is regulated by Mef2c. Fold change in luciferase activity driven by approximately 1 kb of the Crtl1 promoter in the pGL3 luciferase reporter vector was assayed in fetal chicken VICs (A and B) and in NIH3T3 cells (C and D). In fetal chicken VICs (A) and NIH3T3 cells (C), Crtl1 promoter activity was significantly increased with increasing concentrations of Mef2c. (B) Crtl1 promoter activity in the presence of 100 ng Mef2c with the addition of 200 ng of the Mef2-Engrailed dominant negative expression construct resulted in an approximately 30% reduction in Crtl1 reporter activity. (D) Mutations were introduced into the Crtl1 promoter construct at Mef2 Site 1 and Mef2 Site 2 (Crtl1-Mutant 1 and Crtl1-Mutant 2 respectively). Crtl1-Mutant 1 results in an approximately 30% reduction in Crtl1 promoter activation in the presence of 100 ng of Mef2c and Crtl1-Mutant 2 results in an approximately 50% reduction of Crtl1 activity.(*p<0.05, #p<0.1).

To demonstrate that the Mef2 binding sites identified in the Crtl1 promoter are not only able to bind to Mef2c, but that this interaction also results in the regulation of Crtl1 transcription, approximately 1 kb of the wildtype mouse Crtl1 promoter (−979 to +26) was cloned into a pGL3 Basic luciferase reporter vector. This Crtl1 promoter construct was transfected into chicken HH40 mitral VICs and NIH3T3 cells along with a mouse Mef2c expression construct. The luciferase activity of the Crtl1 promoter was measured and in both cell types was found to increase significantly and in a dose dependent manner (Figure 5A,C). A Mef2-Engrailed expression construct, which functions as a dominant-negative repressor of all Mef2 transcription factors [15], was also transfected into the mitral VICs in the presence of 100 ng Mef2c. In response to Mef2-Engrailed, Crtl1 promoter activity was reduced by approximately 30% (p = 0.03) relative to the Crtl1 promoter in the presence of 100 ng of exogenous Mef2c alone, indicating that Crtl1 reporter activity has some dependence on Mef2c expression (Figure 5B).

To further test the dependence of Crtl1 expression on Mef2c, each Mef2 binding site identified in the Crtl1 promoter was mutated using the same intervening –gcg- mutations that were used in the DNA precipitation assay (Figure 4A). The Mef2 consensus site from −707 to −698 (Mef2 Site 2) was mutated from 5′-ctataaataa-3′ to 5′-ctatagcgaa-3′ (Crtl1-Mutant 2) and the Mef2 consensus site (Mef2 Site 1) from −922 to −913 was mutated from 5′-ttataaataa-3′ to 5′-ttatagcgaa-3′ (Crtl1-Mutant 1). Mutation of Mef2 Site 2, results in a diminished response with an approximately 50% reduction in Crtl1 promoter activity at basal levels (p = 7.0×10^−6^). The reduced promoter activity remained in the presence of both 50 ng and 100 ng of exogenous Mef2c (p = 0.0007 and p = 0.014 respectively) (Figure 5D). Mutation of Mef2 site 1, however, only results in an approximately 25% reduction in Crtl1 promoter activity at basal levels (p = 0.084) and in the presence of high concentrations of Mef2c (p = 0.008) (Figure 5D). This data further validates that Crtl1 activity is dependent on Mef2c interaction with the promoter.

## Discussion

The development of the atrioventricular valves begins with the swelling of the endocardial lining of the AV junction and the formation of the AV cushions. The cushions then become populated by endocardially derived cells resulting from endocardial-to-mesenchymal transition (EMT). At this point in time, the endocardial cushion mesenchyme contains a variety of ECM components, including Crtl1, hyaluronan, and versican. These ECM components play important roles in cushion development [2], [31]–[33]. In previous work, we have shown that Crtl1 is widely expressed in the endocardial cushions and AV valves during stages of mesenchymal cell proliferation and differentiation [2]. As the valves elongate and mature, the composition of the ECM changes. This change is characterized by an increase in expression of proteins such as fibronectin and collagen-1 [34] whereas the expression of Crtl1 becomes largely restricted to the atrial and ventricular aspects of the valve leaflets [12].

While there is an increasing knowledge of the role of ECM in valvulogenesis, relatively little is known regarding the spatiotemporal regulation of the ECM during cardiac development. Previously, it was demonstrated that Mef2c expression is found in the cardiomyocytes of the developing heart where it is considered pro-myogenic as it is known to regulate contractile-proteins such as cardiac alpha-actin and alpha-myosin heavy chain [16]–[18]. Here we show that Mef2c expression is not restricted to the myocardium and that it is also found in the endocardium and endocardially-derived mesenchyme of the AV valves from mid to late gestation where it is co-expressed with Crtl1, suggesting that Mef2c may act as an important regulator of Crtl1 expression during cardiac development.

Providing further indication that Mef2c could be involved in the transcriptional regulation of Crtl1, we found that the Crtl1 promoter contains two Mef2 transcription factor binding sites that are conserved between human, mouse, and rat. Testing the hypothesis that these Mef2 binding sites indeed can bind Mef2c and activate Crtl1 transcription, we performed DNA affinity precipitation, ChIP analysis, and luciferase assays. Combined, these *in vitro* and *in vivo* studies provided evidence that Mef2c binds to the Crtl1 5′UTR and activates Crtl1 transcription. To ascertain the importance of each Mef2 binding site, we mutated these two sites and evaluated the Crtl1 promoter response to exogenous Mef2c. Mutation of the Mef2c binding site at −707/−698 resulted in reduction of Crtl1 promoter activity both in the presence and absence of exogenous Mef2c protein, while mutation of the Mef2c binding site at −923/−913 only resulted in a slight reduction of Crtl1 promoter activity. The Crtl1 promoter fragment that was used in these experiments also contained a previously described Sox9 binding site that has been demonstrated to be important in the regulation of Crtl1 in cartilage and bone formation [10], [30]. In the developing bone, Sox9 and Mef2c have been shown to activate the Col10a1 promoter independently or co-activate Col10a1 in an additive fashion [13], [15]. Deletion of either the Sox9 binding site or the Mef2c binding site in the Col10a1 promoter results in a reduction in Col10a1 activation. However deletion of both Sox9 and Mef2c binding sites are needed to result in complete abolishment of promoter activity [15]. It is therefore possible that Crtl1, an important ECM component in developing bone as well as valves, may be similarly regulated and that mutation of the Sox9 binding site within the Crtl1 promoter may be needed to achieve a complete loss of promoter activity.

As described above, during the process of valve remodeling, Crtl1 expression becomes restricted as the mesenchyme within the valves becomes condensed. Tgfβ2 has been demonstrated to be necessary for the repression of Crtl1 during late stages of valve development in order to prevent ectopic differentiation into a cartilage-lineage [35], while Sox9 has been shown to be critical for Crtl1 expression during the early stages of cushion and valve development [12]. Based on expression patterns of Mef2c, its binding of the Crtl1 promoter, and its activation of Crtl1 transcription, we hypothesize that Mef2c could, in addition to its involvement in the regulation of the myogenic gene program, also be involved in the regulation of ECM synthesis in endocardial and endocardially-derived mesenchyme by playing a role in the regulation of Crtl1 expression and valve remodeling.

Mef2c knockout mice die around ED9.5 of development due to inability of the heart to loop and myocardialize [19]. Interestingly, mice that do not express the Crtl1 binding partners versican or hyaluronan, die at early embryonic stages (approximately ED9.5) due to failure of AV cushion formation [32], [36]. Although Mef2c knockout mice fail to form endocardial cushions and have reduced amounts of ventricular cardiac jelly [19], it has yet to be determined whether this is associated with impaired Crtl1 expression in the developing heart.

Proper synthesis, deposition, and alignment of ECM during cardiac valve development is critical for the formation and functionality of the mature leaflets. Even slight alterations in ECM regulation can have long-term consequences for valve mechanics and can contribute to the pathogenesis of valve disease. Therefore, understanding the mechanisms that regulate Crtl1 expression is not only of importance for the understanding of valve development, but can also be of significance in elucidating the etiology of valve diseases.
